# Validity evaluation of a mock examination including multimedia-assisted items for the Korean Nursing Licensing Examination: a mixed-methods study with a counterbalanced design

**DOI:** 10.3352/jeehp.2026.23.21

**Published:** 2026-07-21

**Authors:** Sujin Shin, Inyoung Lee, Heejeong Kim, Rhayun Song, Jun-Ah Song, Hyun Sook Yi, Janghee Park, Mi Kyoung Yim, Minjae Lee, Subin Yu

**Affiliations:** 1 College of Nursing, Ewha Womans University, Seoul, Korea; 2 Department of Nursing, Dongnam Health University, Suwon, Korea; 3 Division of Health Science, Department of Nursing Science, Baekseok University, Cheonan, Korea; 4 College of Nursing, Chungnam National University, Daejeon, Korea; 5 College of Nursing, Korea University, Seoul, Korea; 6 Department of Education, Konkuk University, Seoul, Korea; 7 Department of Medical Education, College of Medicine, Kangwon National University, Chuncheon, Korea; 8 Korea Health Personnel Licensing Examination Institute, Seoul, Korea; Hallym University, Korea

**Keywords:** Nursing education, Educational measurement, Nursing licensure, Psychometrics, Multimedia, Republic of Korea

## Abstract

**Purpose:**

This study aimed to evaluate the validity of multimedia-assisted items (MAIs) in a mock examination by examining their psychometric properties and nursing students’ perceptions.

**Methods:**

This methodological study used a within-group counterbalanced design in which fourth-year nursing students in South Korea completed an online mock examination consisting of paired text-based items and MAIs. Psychometric properties were examined using classical test theory and item response theory, and learner perceptions and response processes were explored through post-examination surveys and focus group interviews.

**Results:**

Among 515 participants, MAIs demonstrated overall difficulty and discrimination comparable to those of text-based items in both classical test theory and item response theory analyses, supporting measurement equivalence across item formats. Post-test surveys indicated that participants perceived MAIs as realistic reflections of clinical practice and as more helpful for problem solving and ability assessment than text-based items. Qualitative findings from focus group interviews (n=15) further supported the educational value of MAIs for assessing clinical judgment while emphasizing the need for appropriate multimedia use. An order effect was also observed: presenting text-based items first led to higher item response theory discrimination parameters in subsequent MAIs (P=0.020).

**Conclusion:**

MAIs demonstrated psychometric properties comparable to those of text-based items and were positively perceived as valid tools for assessing clinical judgment and practical competence. These findings support the feasibility of incorporating multimedia-based assessment into the Korean Nursing Licensing Examination as part of a rigorous computer-based testing framework.

## Graphical abstract

[Fig f6-jeehp-23-21]

## Introduction

### Background/rationale

To reduce the gap between theory and practice in nursing education and support newly licensed nurses’ transition into clinical practice, it is essential to enhance clinical judgment and practical competence [1]. However, these competencies are difficult to assess adequately using written examinations alone. For example, memorizing abnormal breath sounds does not ensure the ability to auscultate lung sounds accurately or make clinical judgments in real situations. Accordingly, simulation-based and technology-enhanced educational approaches have been increasingly adopted in nursing education [2,3]. In contrast, systematic approaches to developing and validating assessment tools that reflect these competencies, particularly those incorporating technology-based item formats, remain limited.

In South Korea, the Korean Health Personnel Licensing Examination Institute (KHPLEI) has established task forces, plans to revise relevant regulations, and intends to implement a phased rollout with the goal of introducing a computer-based testing (CBT) format for the Korean Nursing Licensing Examination (KNLE) by 2028 [4]. The transition to CBT represents not only a change in test delivery but also an opportunity to use diverse item formats that better reflect real clinical situations. This transition may enhance job-related validity and improve item discrimination and reliability through the development of more valid test items. In addition, computer-based assessment allows the use of multimedia, such as videos, audio, and high-resolution images, that are difficult to implement in paper-based tests, thereby diversifying item presentation formats [5]. Multimedia-assisted items (MAIs) can represent authentic problem situations more effectively and convey meaning more efficiently than text alone, enabling more accurate measurement of learning outcomes [6]. Previous studies have also reported improvements in knowledge and performance when multimedia materials were used in education and assessment, supporting the effectiveness of computer-based assessment [7].

The United States nursing licensure examination (National Council Licensure Examination for Registered Nurses) uses computerized adaptive testing (CAT), in which item selection is based on examinees’ responses. Recognizing clinical judgment as a core competency for newly licensed nurses, the National Council of State Boards of Nursing launched the Next Generation NCLEX project and developed the Clinical Judgment Measurement Model as a systematic assessment framework [8]. In South Korea, CBT has also been introduced into national health professional licensure examinations, beginning with the medical licensing examination in 2022 and expanding to other health professions in 2023 [9]. These developments highlight the growing need for the KNLE to adopt assessment formats that can evaluate job-related competencies using MAIs.

High-quality items assessing clinical judgment can be selected from existing nursing licensure examination items and used to develop prototypes of case-based MAIs. In a study by Lee and Shin [10], job-based MAIs were developed and validated, and examinees in a mock examination rated these items as more closely related to actual job performance than traditional paper-based items. Similarly, Jang and Suh [11] developed a mobile-based, multimedia-utilized Nursing Competency Evaluation (NCE) system, and the experimental group reported significantly greater effectiveness in terms of convenience, benefits, usefulness, and satisfaction than the control group in a mock examination. These findings suggest that MAIs may enhance job relevance and assessment authenticity in nursing competency evaluation. They also indicate the value of examining the development and preliminary application of MAIs that reflect authentic clinical contexts within the KNLE.

In summary, the transition to CBT provides an assessment environment in which MAIs can be implemented, whereas MAIs offer an item-level approach to representing complex clinical information relevant to nursing practice. MAIs can be conceptualized as items designed to reflect clinical situations and practice-relevant information when assessing clinical judgment competency. Furthermore, estimating psychometric parameters through item response theory (IRT)-based analysis of recent examination items, alongside the development and pilot testing of multimedia items, is essential for evaluating the feasibility of CAT.

Therefore, this study aimed to administer a mock examination including MAIs and evaluate their validity by analyzing the examination results. Ultimately, the findings are intended to provide foundational evidence to support the transition to a computer-based KNLE.

### Objectives

The purpose of this study was to administer a mock examination including MAIs and paired text-based items with identical content to nursing students and to evaluate the validity of the MAIs by analyzing the examination results in preparation for the transition to a computer-based KNLE.

## Methods

### Ethics statement

This study was conducted in accordance with research ethics principles and was approved by the Institutional Review Board (IRB) of the authors’ affiliated institution (IRB no., ewha-202511-0004-01). Informed consent was obtained from all participants before participation. Focus group interviews were audio-recorded with participants’ consent and transcribed verbatim, and the audio files were permanently deleted immediately after transcription. The study was conducted without compromising participant safety, and all collected data were used solely for research purposes and anonymized to protect participants’ privacy.

### Study design

This study used a methodological design with a within-group counterbalanced approach. Two mock examination formats were administered to nursing students, and psychometric item characteristics, survey responses, and focus group interview data were collected and analyzed. A mixed-methods approach was adopted to obtain both psychometric evidence and response-process validity evidence. Quantitative analyses were used to examine the measurement properties of MAIs compared with text-based items, whereas survey and focus group interview data were used to explore examinees’ perceptions, cognitive responses, and problem-solving experiences. The findings were integrated during interpretation to evaluate MAIs in terms of psychometric performance, clinical authenticity, and examinee response processes.

### Development of multimedia items

The multimedia items used in this study were developed through a stepwise development and validation process before the mock examination. First, focus group interviews and an item development workshop were conducted with 15 nursing faculty members to review items from the 61st to 63rd KNLEs (2021–2023) and identify items suitable for transformation into multimedia formats. Next, a priority evaluation was conducted with 10 nursing faculty members, resulting in the selection of 29 items identified as high priorities for MAI development. Five similar items, defined as those sharing identical or highly similar materials and overlapping core concepts or correct answers, were excluded after consultation with a subject matter expert. Five new items were additionally developed to include newly created MAIs beyond transformed existing KNLE items, yielding a final set of 29 items. To examine content validity, 10 newly licensed nurses evaluated each item using a 4-point Likert scale on 2 dimensions: (1) whether the MAI better assessed job-related competence compared with the text-based item and (2) whether the MAI better reflected clinical practice. The content validity index (CVI) was calculated as the proportion of responses rated 3 or 4, with a CVI of ≥0.78 considered acceptable. All items achieved a CVI of 1.00 for clinical practice reflection. For job-related competence assessment, 27 of the 29 items achieved a CVI of ≥0.78; the remaining 2 items yielded a CVI of 0.70. After an intensive research team meeting, these 2 items were retained in the final item set because their inclusion was not expected to substantially affect overall examination feasibility. A pilot test was conducted with 10 nursing students to assess item clarity. Each item was rated on a 4-point Likert scale for difficulty in understanding or solving the item. All 29 items had mean scores ranging from 1.10 to 1.80 (mean=1.34±0.62), with no item exceeding 2.00, indicating that examinees experienced no substantial difficulty. Based on qualitative feedback, improvements were made to multimedia elements, including image resolution, subtitles, and audio quality.

### Setting

This study was conducted in an online mock examination environment with nursing students in South Korea. Participants were recruited after IRB approval beginning on October 29, 2025, and the mock examination was administered from November 20 to 25, 2025. All examinees participated in the mock examination once using a personal computer during the designated testing period. A post-examination survey was conducted immediately after the mock examination, and focus group interviews were conducted online with 2 groups on November 28, 2025.

### Participants

The sample size was determined based on IRT requirements, with at least 500 participants recommended for 2-parameter logistic (2PL) models [12]. Accounting for a 10% attrition rate, 556 fourth-year nursing students in South Korea were recruited. Eligible participants were those able to complete an online examination. Data from 515 participants were included in the final analysis after excluding 24 non-examinees and 17 cases with invalid responses, defined as a completion time of less than 5 minutes (n=5) or an incomplete examination (n=12). For response-process validity, focus group interviews were conducted with 15 participants, consistent with prior recommendations for achieving data saturation [13]. Among participants who agreed to participate in the focus group interviews, 15 were randomly selected for the qualitative component using a random number table generated in Microsoft Excel (Microsoft Corp.). The 15 participants were divided into 2 focus groups, with 7 participants in Group A and 8 in Group B.

### Study procedure and data collection

**Fig. 1.**
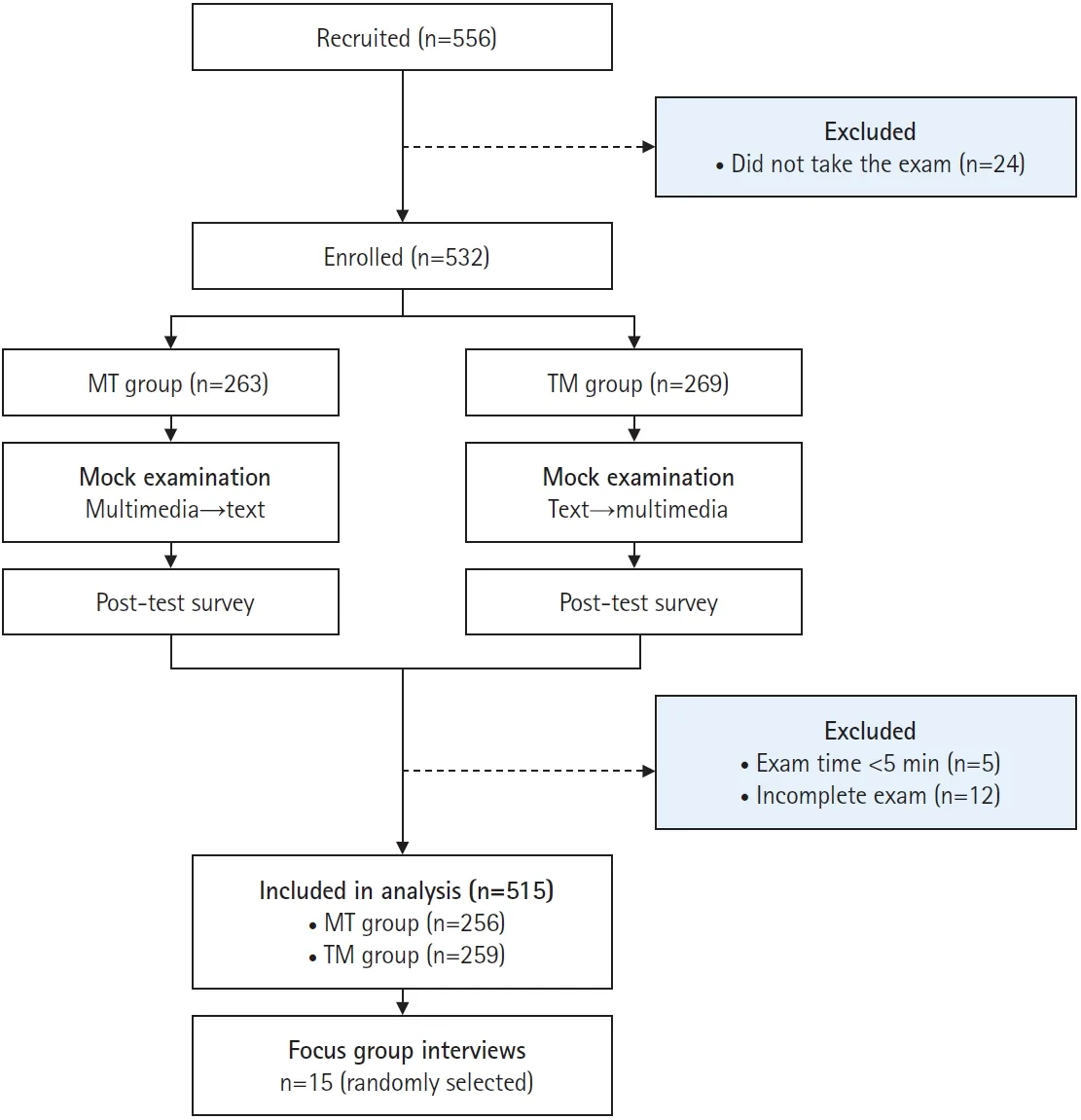
Flow diagram of participant enrollment and data collection

The flow diagram of participant enrollment and data collection is shown in Fig. 1. Participants were randomly assigned to either the MT group (MAIs first, then text-based items) or the TM group (text-based items first, then MAIs). Each format consisted of 29 paired items with identical content, for a total of 58 items. Binary item response data from the mock examination are provided in Dataset 1. Item difficulty and discrimination were analyzed using classical test theory (CTT) and IRT. A post-examination survey assessing perceptions of MAIs, consisting of 6 items on a 5-point Likert scale, was administered after the examination. The survey items examined participants’ perceptions of MAIs in terms of (1) realistic reflection of clinical practice, (2) helpfulness of the presented materials for problem solving, (3) perceived accuracy in assessing examinee ability compared with text-based items, (4) difficulty maintaining concentration while solving MAIs, (5) sufficiency of the allotted time for interpreting and responding to items, and (6) satisfaction with the screen layout. These items were developed by the research team based on the study purpose and the key characteristics of MAIs, including clinical authenticity, use of multimedia materials, perceived assessment accuracy, cognitive burden, response time, and interface usability. The survey questionnaire is provided in Supplement 1, and participant characteristics and post-survey data are provided in Dataset 2. Focus group interviews were conducted with consenting participants to examine response-process validity using a semi-structured interview guide. The focus group interview guide is provided in Supplement 2.

### Bias

To minimize testing-environment bias, all participants completed the mock examination using the same online platform under identical conditions. Item order was randomized within each test format to reduce potential memory and order effects. During qualitative data collection, the researcher maintained a neutral stance and ensured that participants’ experiences were adequately reflected.

### Data analysis

Data analyses were conducted using IBM SPSS Statistics ver. 30.0 (IBM Corp.), Microsoft Excel, and R ver. 4.4.1 (The R Foundation for Statistical Computing). Descriptive statistics were used to analyze participants’ characteristics and perceptions of MAIs. Item difficulty and discrimination were estimated based on CTT and IRT, with the 2PL model applied for IRT analyses using the irtQ package in R. CTT-based discrimination was calculated using the 27% upper–lower group method and corrected item–total correlations. Pearson correlation analyses were conducted to examine the consistency of the 5 item parameters between paired MAIs and text-based items. MAIs and text-based items were also analyzed as 2 separate tests. Total scores were calculated for the 29 MAIs (M01–M29) and the 29 text-based items (T01–T29), respectively, and IRT ability parameters were estimated using the 2PL model. Pearson correlations were then computed between the total scores and between the ability parameters derived from the 2 formats. Total scores and ability parameters were compared between the MT and TM groups using independent-samples t-tests. Item parameters estimated separately for each group were compared using paired-samples t-tests and Pearson correlation analyses. Quantitative and qualitative findings were integrated during interpretation to relate psychometric item characteristics to participants’ experiences and response processes.

Qualitative data were analyzed using inductive content analysis. Audio-recorded interviews were transcribed verbatim and repeatedly read to obtain an overall understanding of the data. Two researchers independently coded the transcripts, compared their codes through discussion, and resolved discrepancies by consensus. Codes with similar meanings were grouped into subcategories, which were then organized into broader categories and themes. To enhance trustworthiness, researcher triangulation, repeated transcript review, and consensus meetings were used throughout the analytic process. The focus group interviews were facilitated by nursing faculty members on the research team, none of whom had a prior relationship with the participants in their assigned group. To minimize potential power imbalances, interviewers were deliberately assigned based on participants’ regional affiliations, ensuring that no interviewer conducted interviews at an institution where the participants were enrolled. Member checking was not performed because of logistical constraints and the exploratory nature of the qualitative component. Transcripts were not returned to participants for review; however, credibility was supported through clarification questions during the interviews and team-based review of the transcripts and themes.

## Results

### Participants

Among the 515 participants, 256 (49.71%) were assigned to the MT group and 259 (50.29%) to the TM group. Most participants were female (85.44%), and the mean age was 24.68±5.51 years. The largest proportion of participants was from the Busan–Ulsan–Daegu–Gyeongsang region (24.08%). Before the mock examination, 31.26% of participants had experience with examinations including MAIs. Images were the most commonly used media type (90.06%), followed by audio (50.93%) and video (24.84%) (Table 1).

### Characteristics of the developed MAIs

**Fig. 2.**
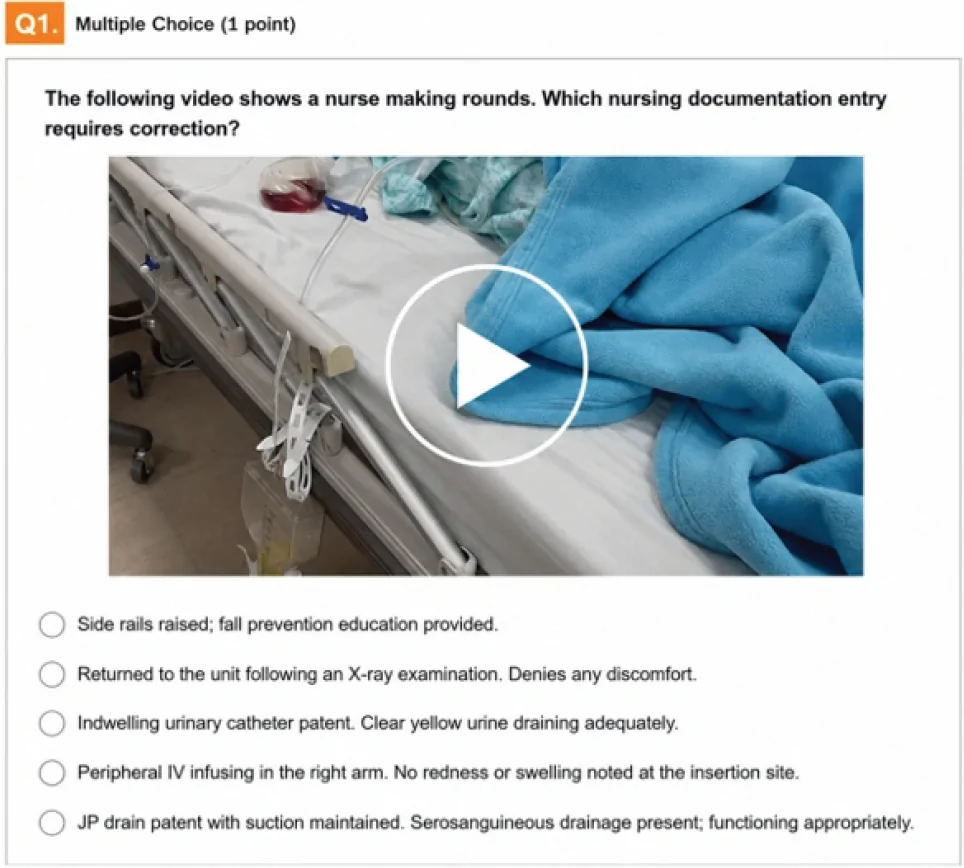
Example of multimedia-assisted item used in the mock examination

Of the 29 developed MAIs, adult nursing accounted for the largest proportion (n=10; 34.48%), followed by pediatric nursing (n=9; 31.03%), maternal nursing (n=6; 20.69%), fundamental nursing (n=3; 10.34%), and psychiatric nursing (n=1; 3.45%). By knowledge level, problem-solving items were most common (n=15; 51.72%), followed by interpretation (n=12; 41.38%) and recall (n=2; 6.90%). By nursing process, assessment items were most frequent (n=13; 44.83%). Images were used to present visual patient-related information, such as pressure ulcer status, patient positioning, and diagnostic test results. Audio materials provided auditory clinical cues, including lung sounds and statements from patients or nurses. The videos depicted dynamic clinical situations, such as electrocardiogram rhythms, chest drainage bottle status, and patients’ physical symptoms or clinical signs. These materials were designed to provide clinical cues and contextual information that are difficult to convey through text alone. The characteristics of all items are summarized in Table 2, and an example of the examinee test screen is shown in Fig. 2.

### Psychometric properties of the items

Psychometric analyses were conducted based on paired items with identical content, and all participants (N=515) were pooled to minimize the effects of test-format presentation order.

#### Classical test theory

In the CTT-based item analysis, the correct response rate for MAIs ranged from 20.58% to 98.06%, with a mean of 63.37%±21.57%. Discrimination index 1, calculated using the upper–lower group method, ranged from 0.04 to 0.63 (mean=0.29±0.14), and discrimination index 2, based on item–total correlation, ranged from 0.03 to 0.40 (mean=0.21±0.08). For text-based items, the correct response rate ranged from 26.41% to 99.22%, with a mean of 64.67%±19.84%. Discrimination index 1 ranged from 0.01 to 0.63 (mean=0.30±0.15), and discrimination index 2 ranged from 0.07 to 0.41 (mean=0.22±0.08). The CTT-based item analysis results for all items are presented in Table 3.

#### Item response theory

In the IRT-based item analysis, the difficulty parameters of MAIs ranged from –4.59 to 27.35 (mean=–0.18±5.63), and discrimination parameters ranged from 0.05 to 1.49 (mean=0.67±0.34). For text-based items, difficulty parameters ranged from –5.01 to 6.38 (mean=–1.12±2.24), and discrimination parameters ranged from 0.14 to 1.62 (mean=0.69±0.34). However, several items showed extreme difficulty estimates (|b|>4) with large standard errors (>2), indicating some instability in parameter estimation. This instability may be related to the sample size for items with extreme values. The IRT-based item analysis results for all items are presented in Table 4.

**Fig. 3.**
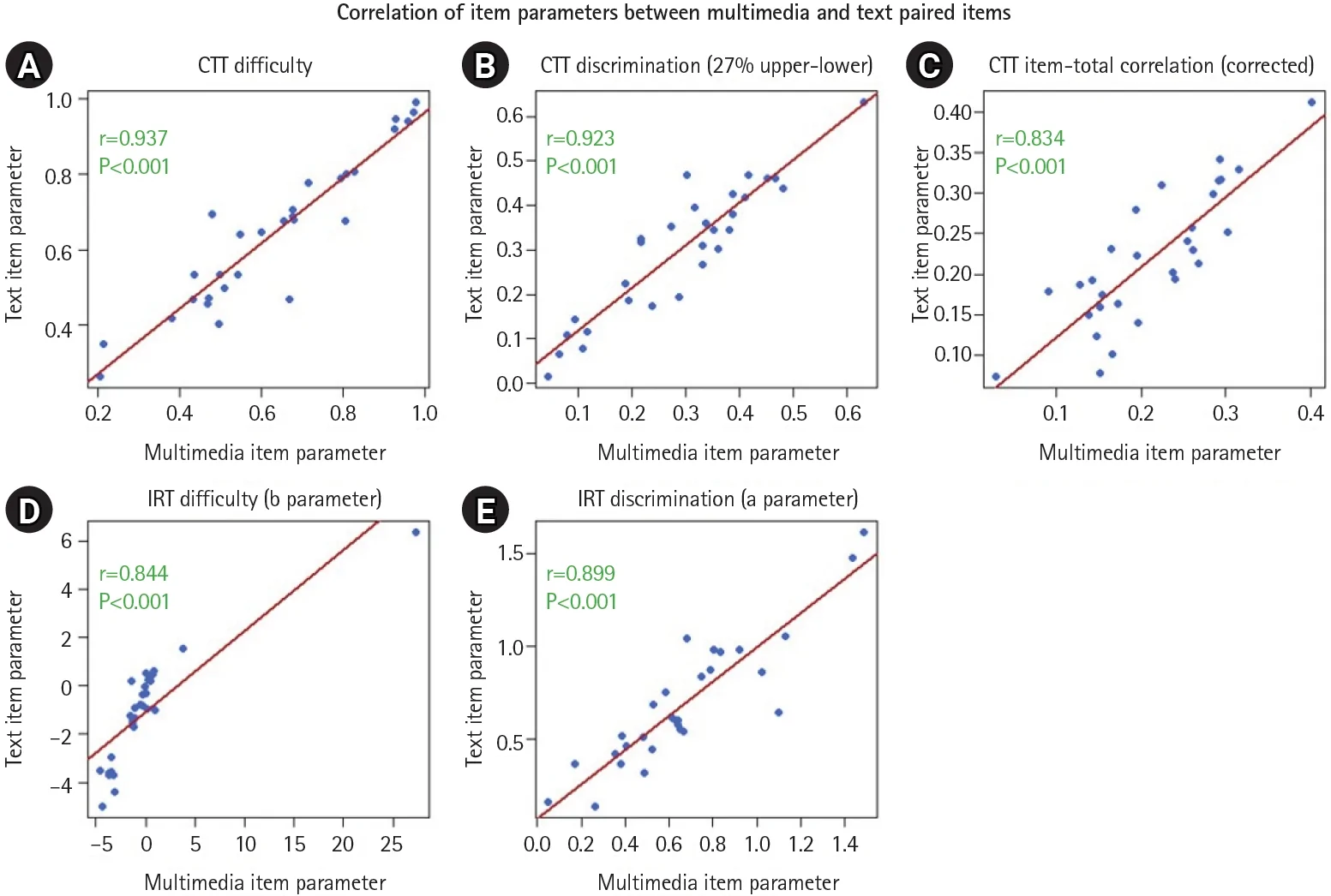
(A–E) Scatter plots of item parameter correlations between paired multimedia-assisted and text-based items

Pearson correlation analyses were conducted to evaluate the consistency of item parameters between MAIs and their corresponding text-based items (Supplement 3). Strong positive correlations were observed across all 5 item parameters. Correlation coefficients were 0.94 for CTT difficulty, 0.92 for CTT discrimination using the upper–lower discrimination index, 0.83 for corrected item–total correlation, 0.84 for the IRT difficulty parameter, and 0.90 for the IRT discrimination parameter; all were statistically significant (P<0.001). Scatter plots of item parameter correlations between paired multimedia-assisted and text-based items are presented in Fig. 3.

#### Correlation between multimedia-assisted and text-based item formats

**Fig. 4.**
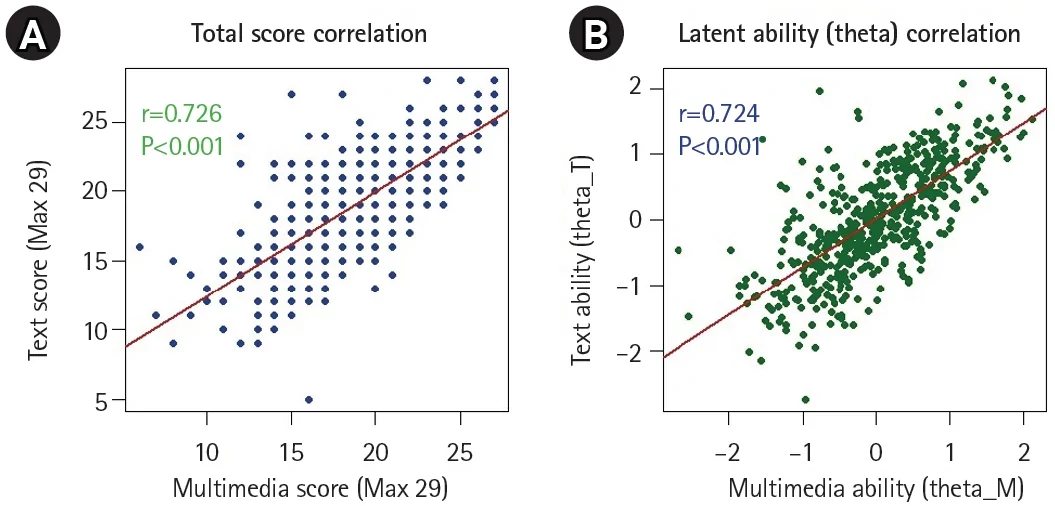
Scatter plots of total score correlation (A) and latent ability correlation (B) between multimedia-assisted and text-based item formats.

MAIs and text-based items were analyzed as separate tests to examine the correlations between total scores and ability parameters derived from each format. Total scores were strongly correlated between the 2 formats (r=0.73, P<0.001), and ability parameters also showed a strong correlation (r=0.72, P<0.001). Scatter plots illustrating these correlations are presented in Fig. 4.

#### Group comparison between the MT and TM groups

**Fig. 5.**
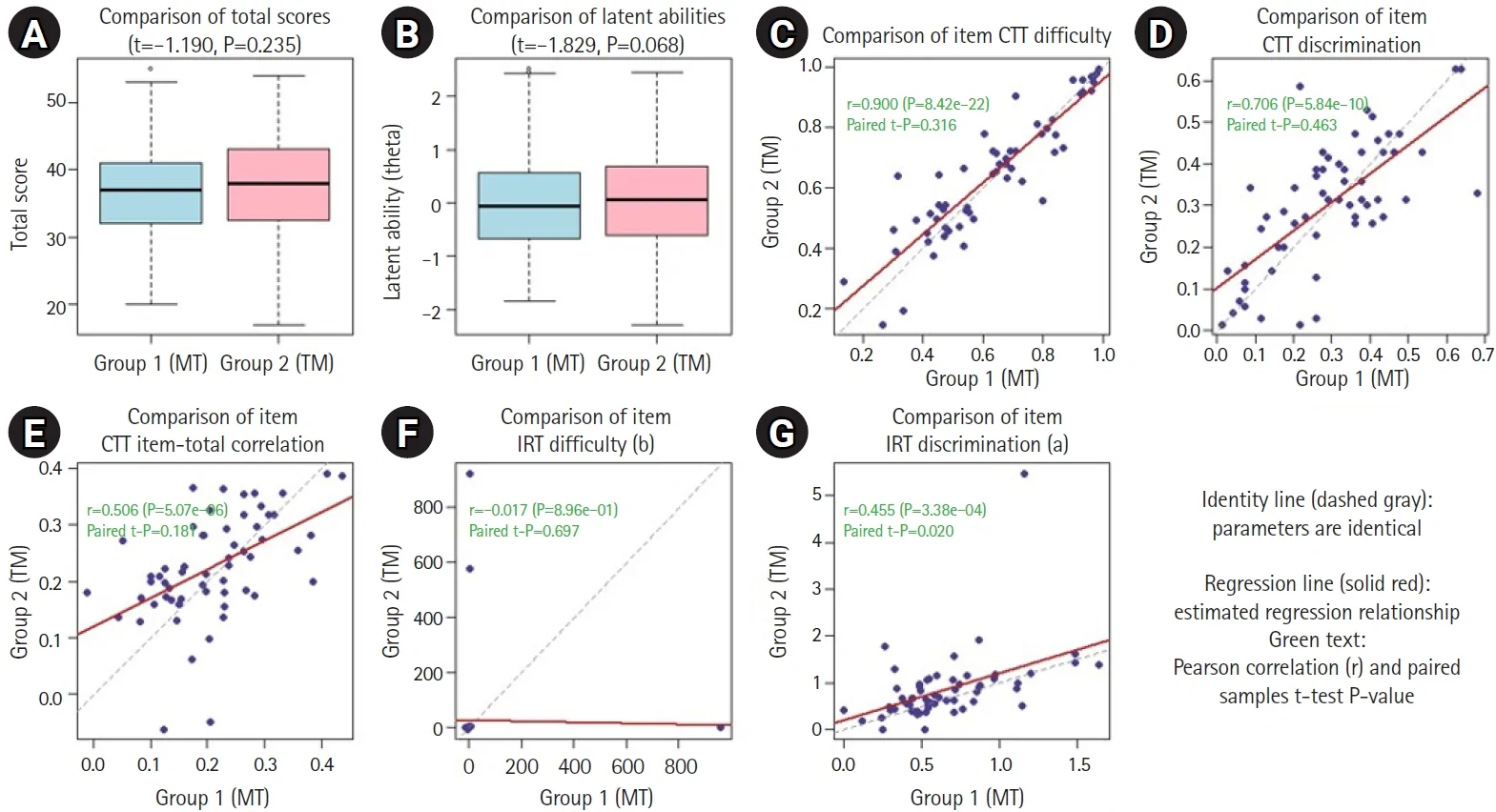
(A–G) Comparison of total scores, latent abilities, and item parameters between the 2 groups. MT group: multimedia-assisted items (MAIs) first, then text-based items; TM group: text-based items first, then MAIs. CTT, classical test theory; IRT, item response theory.

Independent t-tests showed no statistically significant differences between the MT and TM groups in total scores or ability parameters (Supplement 4). The mean total score was 36.76±6.87 in the MT group and 37.49±7.10 in the TM group (t=–1.19, P=0.235). The mean ability parameter was –0.07±0.88 in the MT group and 0.07±0.92 in the TM group (t=–1.83, P=0.068). Item parameters were estimated separately for the MT and TM groups (Supplement 5). No statistically significant differences were observed in CTT difficulty, CTT discrimination, corrected item–total correlation, or IRT difficulty. However, the IRT discrimination parameter was significantly higher in the TM group than in the MT group (t=–2.39, P=0.020). Pearson correlations between the MT and TM groups ranged from –0.02 to 0.90 across item parameters. These correlations are illustrated in Fig. 5. R script for item analysis using the irtQ package and data analysis outputs are presented in Supplement 6.

### Post-test survey results

Participants rated MAIs as realistically reflecting clinical practice (4.13±0.74) and reported that the presented materials were helpful for problem solving (4.01±0.84). They also perceived MAIs as more accurately assessing examinee ability than text-based items (3.85±0.95). Participants reported no major difficulty maintaining concentration while solving MAIs (3.74±1.10) and indicated that the allotted time for interpreting and responding to items was sufficient (4.34±0.68). Satisfaction with the screen layout was relatively high (4.09±0.94). A total of 67 participants (13.01%) reported that some items included content they had not previously learned (Table 5).

### Qualitative findings from focus group interviews

Fifteen participants were divided into 2 groups, and 2 focus group interviews were conducted. All participants were female, with a mean age of 25.5 years. Four participants (26.7%) had prior experience with MAIs. Group A consisted of 7 participants aged 22–39 years from the Gyeongsang, Gyeonggi, Gangwon, and Jeolla regions, including 2 participants with prior MAI experience. Group B consisted of 8 participants aged 22–30 years from the Jeolla, Seoul, Chungcheong, Gyeongsang, and Gangwon regions, including 2 participants with prior MAI experience. Each group was homogeneous in that all participants were fourth-year female nursing students, whereas heterogeneity between and within groups was considered by including participants from different regions and with varying prior experience with MAIs. Qualitative analysis yielded 3 themes, 8 categories, and 32 subcategories (Table 6). The 3 themes were (1) learners’ initial experiences and cognitive responses to MAIs, (2) experiences of assessing practical competence reflecting real clinical settings, and (3) the educational value and appropriate use of multimedia-based assessment.

#### Learners’ initial experiences and cognitive responses to MAIs

Participants experienced unfamiliarity, confusion, and tension when they first encountered MAIs, while also perceiving them as a novel item format. They tended to find MAIs difficult because they differed from conventional text-based items and required them to interpret patient information resembling real clinical situations. Participants also reported difficulty applying theoretical knowledge to materials that reflected real clinical contexts. In addition, they felt burdened by multi-source information processing because they had to observe several elements on the screen simultaneously, replay materials to confirm missed information, and spend more time solving items. Low-realism materials, including some AI-generated videos, were also reported to disrupt concentration.

#### Experiences of assessing practical competence reflecting real clinical settings

Participants perceived that MAIs required different problem-solving strategies and higher-order thinking processes compared with text-based items. Rather than relying on characteristic textual cues or fragmentary knowledge, they had to integrate various types of clinical information, such as the patient’s condition, sounds, and vital signs, and determine priorities. Participants viewed MAIs as suitable for practice-based assessment because they clearly represented clinical situations and required assessment and judgment processes similar to those used in actual clinical practice. They also reported that simulation and clinical training experiences facilitated problem solving in MAIs.

#### The educational value and appropriate use of multimedia-based assessment

Participants recognized the educational value and learning effects of MAIs, including intuitive comprehension of complex concepts, faster problem solving, and increased immersion during assessment. They emphasized the need to expand multimedia-based learning and suggested developing standardized multimedia educational materials through schools or professional associations. However, they also noted that multimedia materials are not necessary for all items and that text-based items and MAIs should be used in a complementary manner. Some participants pointed out that the advantages of MAIs were not fully realized when items could be solved without engaging with the multimedia materials.

## Discussion

### Key results

This study administered a mock examination presenting identical item content in both text-based and multimedia formats to examine psychometric characteristics by item type and assess learners’ perceptions of MAIs, thereby evaluating the validity of MAIs. The study used a within-group counterbalanced approach to examine the characteristics of MAIs rather than determine their superiority over conventional items.

CTT-based item analyses showed that the overall mean correct response rate was comparable between MAIs and text-based items; however, the direction of the difference varied across individual items. For items in which MAIs showed lower correct response rates, this may reflect the additional difficulty of connecting learned theoretical knowledge with multimedia-presented information. Lee and Shin [10] reported that participants found it difficult to combine theoretical content with multimedia-presented information and were not accustomed to integrating practical experience with theoretical knowledge when solving MAIs. However, several items demonstrated higher correct response rates in the multimedia format. For example, items presenting fetal/newborn reflexes and fundal height using images or videos (Items 3, 8, and 16) showed good discrimination (all>0.30), with higher correct response rates for multimedia than for text-based versions. These findings suggest that the mode of information presentation, text versus multimedia, can influence item difficulty and that multimedia, particularly audiovisual materials that convey critical information, may reduce extraneous cognitive load and facilitate problem understanding. This aligns with prior research indicating that MAIs support situational understanding and problem solving [10]. Overall, discrimination indices were similarly distributed across multimedia and text-based items, with mean values of approximately 0.3 for both formats. Compared with the KNLE, the upper–lower index of MAIs in this study (0.29) was higher than that of text-based items in the 61st–65th KNLEs (0.18–0.21), whereas the item–total correlation of MAIs (0.21) was slightly lower than that of KNLE text-based items (0.23–0.24). Taken together, these findings suggest that MAIs provided acceptable levels of discrimination and could be used alongside text-based items to differentiate examinee ability.

In the IRT-based item analysis, the mean discrimination parameters were similar between MAIs and text-based items, indicating that both formats had a comparable capacity to differentiate examinees. MAIs, however, tended to be estimated as more difficult than text-based items, and several items showed extreme difficulty estimates (e.g., |b|>4) accompanied by large standard errors, indicating instability in parameter estimates for these items. This instability suggests that the sample size may have been insufficient for stable parameter estimation of items with extreme difficulty, highlighting the need for further validation using larger samples. Moreover, items with extremely low difficulty estimates (Items 9, 11, and 20) had correct response rates exceeding 92%, likely because the multimedia materials directly conveyed visual or auditory cues that facilitated correct responses; this may have limited the extent to which these items could discriminate across the ability range. In contrast, Item 27, which presented a nurse rounding scenario via video, showed a correct response rate of 20.58%, approximating chance level. This may reflect excessive extraneous cognitive load imposed by the simultaneous presentation of multiple clinical cues, compounded by the fact that this item represented a novel format that departed from conventional KNLE item structures.

Across CTT- and IRT-based indices, paired MAIs and text-based items showed strong positive correlations, suggesting that converting items from text to multimedia format did not substantially alter their relative measurement properties within this mock examination. Although absolute difficulty levels differed between formats for some items, the relative ordering of item difficulty and discrimination was largely preserved across the 2 formats. Items that were relatively easy or difficult in text format tended to remain so when presented as MAIs, and items that showed higher discrimination in text format generally retained their discriminative capacity in multimedia format. These findings indicate that introducing MAIs is unlikely to markedly compromise the measurement characteristics established for existing text-based items. When MAIs and text-based items were analyzed as separate tests, total scores and ability parameters were also strongly correlated between the 2 formats. Total scores derived from MAI and text-based item sets were highly correlated (r=0.73), and the corresponding ability parameters showed a similarly high correlation (r=0.72). This suggests that examinee performance was estimated similarly across the MAI and text-based item sets and supports the consistency of ability estimation across the 2 formats.

Regarding the order in which MAIs and text-based items were administered, there were no statistically significant differences between the MT and TM groups in total scores or ability estimates, indicating that format order did not meaningfully affect overall performance. However, the higher 2PL discrimination parameter in the TM group suggests that format order may still have had a small effect on the measurement precision of certain items. Specifically, completing text-based items first may have influenced how some items functioned when participants subsequently answered MAIs. When item parameters were estimated separately for each group, discrepancies in IRT difficulty estimates were mainly observed for items with very low discrimination and extreme b values. These discrepancies are more likely to reflect instability due to limited information for items at the extremes of the ability distribution than true differences in item difficulty between groups.

Post-examination survey results indicated that participants perceived MAIs as realistic reflections of clinical settings and as helpful for problem solving. Participants also reported that MAIs could assess examinee ability more accurately, suggesting that multimedia-based assessment may be an appropriate tool for evaluating clinical judgment and integrative thinking beyond rote knowledge. This finding is consistent with the focus group theme concerning experiences of assessing practical competence reflecting real clinical settings. These findings suggest that the value of MAIs extends beyond psychometric performance to include clinical authenticity, examinee perceptions, and response-process evidence for clinical judgment.

Qualitative analysis further showed that learners perceived MAIs as facilitating a clearer understanding of clinical situations and requiring cognitive processes such as integrating complex information and prioritizing problems. However, some participants noted that excessive multimedia content could increase cognitive load, aligning with cognitive load theory, which emphasizes that the amount and presentation of information influence learning effectiveness [14]. Prior studies have likewise reported that audiovisual materials not directly aligned with learning objectives may increase learners’ cognitive burden [15]. Therefore, rather than being used indiscriminately, MAIs should be applied selectively and used in a complementary manner with text-based items.

These findings suggest that the implementation of MAIs in the computer-based KNLE should follow a progressive approach rather than immediate large-scale adoption. The rationale for introducing MAIs is not that they are psychometrically superior to conventional text-based items but that they can provide comparable measurement performance while enhancing the authenticity of clinical scenarios and supporting the transition to CBT-based examinations. Therefore, MAIs should be used selectively when multimedia presentation adds value to the assessment of clinical judgment, rather than being applied to all item types. At the national examination level, detailed operational standards, item review procedures, and trained item developers are needed to apply standardized MAI development guidelines within the existing item development system. In addition, preparatory systems, such as practice items and tutorials, should be provided to reduce examinees’ cognitive burden and improve their familiarity with MAI formats. Rather than predetermining a fixed proportion of MAIs, it may be more appropriate to first expand data-based items and then progressively introduce a small number of MAIs for items in which multimedia presentation is essential. Future MAI development should also expand beyond static images and figures to include video- and audio-based items that reflect dynamic clinical situations, such as changes in a patient’s condition over time. Further evidence should also be accumulated regarding examinees’ acceptance, cognitive load, response time, item difficulty, discrimination, and test efficiency according to the proportion and types of MAIs.

### Limitations

This study has several limitations. First, instability in parameter estimation was observed for some items with extreme difficulty in the IRT analysis, warranting caution in interpreting the results. Second, because the study was conducted in an online mock examination environment, control over testing time and location was limited, and the conditions may not fully reflect those of an actual examination.

Despite these limitations, the findings provide important implications for applying MAIs to national examinations. Standardized guidelines and review procedures for MAI development are needed. In addition, preparatory strategies to reduce examinees’ initial cognitive burden should be implemented, and a phased introduction with an appropriate balance between multimedia and text-based items is recommended to ensure stable test administration. Future research should conduct item-level analyses to further explore specific items for which text-based items or MAIs yielded statistically higher correct response rates, providing deeper insights into the relative strengths of each item type.

### Conclusion

This study examined the validity of MAIs by analyzing psychometric characteristics and learner perceptions using a mock examination that presented identical item content in text-based and multimedia formats. The findings showed that multimedia items demonstrated overall difficulty and discrimination comparable to those of text-based items, while some items facilitated problem understanding and problem solving by realistically representing clinical situations. Learners perceived multimedia items as reflective of clinical practice and useful for assessing practical competence. These results suggest that MAIs can serve as effective assessment tools for evaluating clinical judgment and integrative thinking in nursing education and provide foundational evidence for improving the quality of assessment in future health professions education.

## Figures and Tables

**Figure f6-jeehp-23-21:**
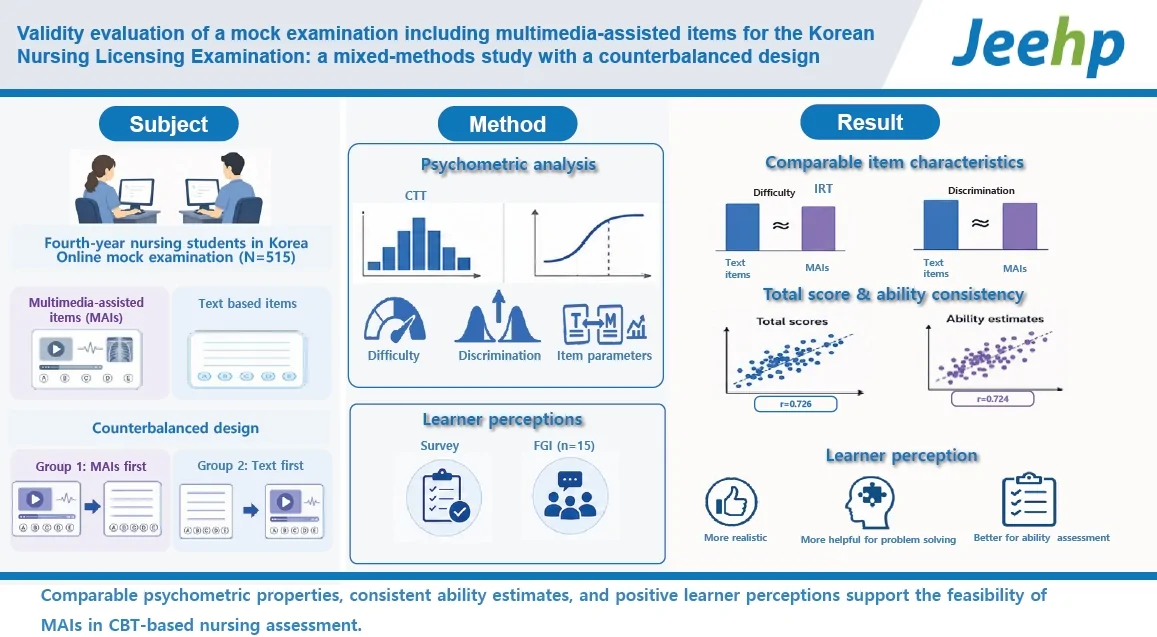


**Table 1.** General characteristics of participants in the mock examination

Characteristic	MT group (n=256)	TM group (n=259)	Total (N=515)
Sex			
Female	221 (86.33)	219 (84.56)	440 (85.44)
Male	35 (13.67)	40 (15.44)	75 (14.56)
Age (yr)	24.83±5.52	24.53±5.50	24.68±5.51
Region			
Seoul	21 (8.20)	27 (10.42)	48 (9.32)
Gyeonggi/Incheon	32 (12.50)	31 (11.97)	63 (12.23)
Gangwon	36 (14.06)	51 (19.69)	87 (16.89)
Busan/Ulsan/Daegu/Gyeongsang	59 (23.05)	65 (25.10)	124 (24.08)
Gwangju/Jeolla	57 (22.27)	42 (16.22)	99 (19.22)
Daejeon/Chungcheong/Sejong	39 (15.23)	29 (11.20)	68 (13.20)
Jeju	12 (4.69)	14 (5.41)	26 (5.05)
Prior experience with multimedia-assisted items			
Yes	79 (30.86)	82 (31.66)	161 (31.26)
Images	70 (88.61)	75 (91.46)	145 (90.06)
Audio	41 (51.90)	41 (50.00)	82 (50.93)
Video	24 (30.38)	16 (19.51)	40 (24.84)
No	177 (69.14)	177 (68.34)	354 (68.74)

Values are presented as number (%) or mean±standard deviation. Multiple responses were allowed for types of multimedia materials. MT group: MAIs first, then text-based items; TM group: text-based items first, then MAIs. MAIs, multimedia-assisted items.

**Table 2.** Characteristics of the 29 multimedia-assisted items

Item no.	Subject	Knowledge level	Nursing process	Multimedia material location	Multimedia material content	Multimedia type
1	Adult	Interpretation	Assessment	Item stem	Pressure ulcer	Image
2	Adult	Interpretation	Diagnosis	Item stem	Breath sounds	Audio
3	Maternal	Interpretation	Assessment	Response options	Fetal presentation	Image
4	Pediatric	Problem-solving	Implementation	Item stem	Cough sounds, breath sounds	Audio
5	Pediatric	Interpretation	Assessment	Item stem	Neonatal breathing pattern	Video
6	Fundamental	Recall	Assessment	Response options	Range of joint motion	Image
7	Maternal	Interpretation	Assessment	Item stem	Speculum examination findings	Image
8	Pediatric	Interpretation	Assessment	Item stem	Neonatal reflexes	Video
9	Pediatric	Problem-solving	Implementation	Item stem	Neonatal milia	Image
10	Pediatric	Problem-solving	Implementation	Item stem	Neonatal status at 1 and 5 minutes	Video
11	Pediatric	Problem-solving	Implementation	Item stem	Airway obstruction in a child	Video
12	Fundamental	Interpretation	Assessment	Response options	Body alignment	Image
13	Adult	Interpretation	Assessment	Item stem	Physical assessment	Video
14	Adult	Problem-solving	Implementation	Item stem	Patient with heart failure	Video
15	Maternal	Interpretation	Implementation	Response options	Positioning to prevent uterine retroversion	Image
16	Maternal	Recall	Assessment	Response options	Fundal height	Image
17	Maternal	Interpretation	Assessment	Item stem	Stages of vaginal delivery	Video
18	Maternal	Problem-solving	Implementation	Item stem	Electronic fetal monitoring graph	Image
19	Adult	Problem-solving	Implementation	Item stem	COPD patient situation/sputum, ABGA results	Video/image
20	Adult	Problem-solving	Implementation	Item stem	Chest X-ray/breath sounds	Image/audio
21	Pediatric	Problem-solving	Implementation	Item stem	Premature infant status	Video
22	Pediatric	Interpretation	Assessment	Item stem	Stool of a child with intussusception	Image
23	Pediatric	Problem-solving	Assessment	Item stem	Face of a child with exotropia	Image
24	Psychiatric	Problem-solving	Implementation	Item stem	Patient with mania	Video
25	Adult	Problem-solving	Implementation	Item stem	Chest drainage bottle	Video
26	Adult	Problem-solving	Implementation	Item stem	Chest drainage bottle	Image
27	Fundamental	Interpretation	Assessment	Item stem	Nurse rounding situation	Video
28	Adult	Problem-solving	Implementation	Item stem	Electrocardiogram	Video
29	Adult	Problem-solving	Implementation	Item stem	Electrocardiogram	Video

COPD, chronic obstructive pulmonary disease; ABGA, arterial blood gas analysis.

**Table 3.** Classical test theory results for multimedia-assisted and text-based items (N=515)

Item no.	Multimedia-assisted items			Text-based items		
	Proportion correct (%)	Upper–lower discrimination index	Corrected item–total correlation	Proportion correct (%)	Upper–lower discrimination index	Corrected item–total correlation
1	47.96	0.39	0.22	69.32	0.42	0.31
2	93.01	0.11	0.15	94.56	0.08	0.12
3	66.80	0.30	0.19	46.99	0.47	0.28
4	65.63	0.33	0.26	67.57	0.31	0.23
5	51.07	0.63	0.40	49.90	0.63	0.41
6	67.77	0.39	0.25	68.74	0.38	0.24
7	60.00	0.47	0.31	64.85	0.46	0.33
8	80.78	0.32	0.30	67.77	0.40	0.25
9	95.92	0.08	0.17	94.17	0.11	0.16
10	82.91	0.19	0.15	80.58	0.22	0.17
11	97.28	0.06	0.14	96.50	0.06	0.15
12	21.36	0.22	0.14	34.95	0.32	0.19
13	71.65	0.35	0.28	77.86	0.35	0.30
14	79.61	0.19	0.15	78.83	0.19	0.16
15	54.17	0.38	0.24	53.59	0.35	0.20
16	49.71	0.45	0.29	40.39	0.46	0.32
17	54.95	0.48	0.29	64.08	0.44	0.32
18	67.96	0.42	0.29	67.96	0.47	0.34
19	43.30	0.41	0.26	46.99	0.42	0.26
20	92.62	0.09	0.13	91.84	0.14	0.19
21	80.97	0.24	0.20	80.00	0.17	0.14
22	67.77	0.33	0.24	70.68	0.27	0.19
23	47.18	0.27	0.16	47.18	0.35	0.23
24	98.06	0.04	0.15	99.22	0.01	0.08
25	43.69	0.29	0.17	53.40	0.19	0.10
26	46.99	0.22	0.09	45.63	0.32	0.18
27	20.58	0.12	0.03	26.41	0.12	0.07
28	49.90	0.34	0.19	53.40	0.36	0.22
29	38.06	0.36	0.27	41.94	0.30	0.21
Mean	63.37	0.29	0.21	64.67	0.30	0.22
SD	21.57	0.14	0.08	19.84	0.15	0.08

SD, standard deviation.

**Table 4.** Item response theory results for multimedia-assisted and text-based items (N=515)

Item no.	Multimedia-assisted items				Text-based items			
	Difficulty (b)	SE	Discrimination (a)	SE	Difficulty (b)	SE	Discrimination (a)	SE
1	0.13	0.19	0.68	0.16	–0.96	0.22	1.05	0.22
2	–4.38	1.95	0.63	0.29	–5.01	2.47	0.60	0.32
3	–1.41	0.39	0.53	0.13	0.19	0.17	0.69	0.14
4	–1.11	0.45	0.64	0.25	–1.32	0.54	0.60	0.24
5	–0.06	0.13	1.44	0.24	–0.01	0.13	1.48	0.25
6	–1.27	0.35	0.64	0.16	–1.46	0.41	0.58	0.15
7	–0.53	0.19	0.92	0.22	–0.76	0.19	0.98	0.23
8	–1.60	0.28	1.10	0.22	–1.26	0.33	0.65	0.16
9	–3.52	1.27	1.02	0.43	–3.58	1.23	0.86	0.34
10	–3.42	1.45	0.48	0.21	–2.93	1.03	0.51	0.19
11	–3.66	1.55	1.13	0.59	–3.60	1.45	1.05	0.50
12	3.78	1.72	0.35	0.16	1.54	0.59	0.42	0.14
13	–1.32	0.29	0.80	0.17	–1.52	0.32	0.98	0.22
14	–3.71	1.84	0.38	0.20	–3.70	1.95	0.37	0.20
15	–0.35	0.26	0.52	0.17	–0.34	0.28	0.45	0.16
16	0.01	0.17	0.79	0.17	0.51	0.17	0.87	0.18
17	–0.31	0.18	0.75	0.16	–0.80	0.21	0.84	0.18
18	–1.04	0.29	0.84	0.23	–0.93	0.23	0.97	0.24
19	0.47	0.33	0.62	0.21	0.21	0.29	0.62	0.21
20	–4.59	2.39	0.58	0.32	–3.50	1.28	0.76	0.30
21	–3.10	1.52	0.49	0.24	–4.41	3.21	0.32	0.23
22	–1.25	0.40	0.65	0.21	–1.69	0.61	0.56	0.21
23	0.30	0.37	0.39	0.17	0.23	0.28	0.52	0.18
24	–3.26	1.03	1.49	0.58	–3.70	2.63	1.62	1.59
25	0.98	0.86	0.26	0.17	–1.00	1.57	0.14	0.17
26	0.71	0.85	0.17	0.13	0.49	0.36	0.37	0.14
27	27.35	79.51	0.05	0.14	6.38	5.71	0.16	0.15
28	0.01	0.45	0.41	0.21	–0.31	0.40	0.47	0.22
29	0.80	0.28	0.66	0.17	0.64	0.31	0.54	0.16
Mean	–0.18	3.48	0.67	0.23	–1.12	0.99	0.69	0.26
SD	5.63	14.64	0.34	0.12	2.24	1.23	0.34	0.27

SE, standard error.

**Table 5.** Participants’ perceptions of multimedia-assisted items in the mock examination (N=515)

Item	No. (%)					Mean±SD
	Strongly disagree	Disagree	Neutral	Agree	Strongly agree	
1. MAIs reflected clinical practice realistically.	3 (0.58)	11 (2.14)	61 (11.84)	280 (54.37)	160 (31.07)	4.13±0.74
2. The multimedia materials provided in the items were helpful for problem solving.	3 (0.58)	25 (4.85)	87 (16.89)	250 (48.54)	150 (29.13)	4.01±0.84
3. MAIs assess examinees’ abilities more accurately than text-based items.	3 (0.58)	46 (8.93)	123 (23.88)	197 (38.25)	146 (28.35)	3.85±0.95
4. I did not experience major difficulty concentrating while solving MAIs.	20 (3.88)	66 (12.82)	77 (14.95)	219 (42.52)	133 (25.83)	3.74±1.10
5. The time provided to interpret and respond to the MAIs was sufficient.	0 (0.00)	10 (1.94)	31 (6.02)	247 (47.96)	227 (44.08)	4.34±0.68
6. I did not experience discomfort when using the screen/interface during the test.	8 (1.55)	29 (5.63)	69 (13.40)	213 (41.36)	196 (38.06)	4.09±0.94

SD, standard deviation; MAI, multimedia-assisted item.

**Table 6.** Themes, categories, and subcategories derived from focus group interviews

Theme	Category	Subcategory
Initial cognitive responses to MAIs	Perceptions of a new item format	Novelty of first exposure to MAIs
		Difficulty adapting to unfamiliar item formats
		Difficulty interpreting patient information similar to real clinical situations
		Difficulty applying theoretical knowledge to materials reflecting real clinical contexts
	Cognitive load during multi-source information processing	Burden of integrating diverse information presented simultaneously
		Difficulty identifying key information among multiple sources
		Increased cognitive load due to time constraints
		Disruption of concentration caused by low-realism materials
Experiences of assessing practice-based competencies reflecting real clinical settings	Need for higher-order thinking processes	Limitations of text-based items in capturing problem-solving strategies
		Requirement for integrating complex clinical information
		Assessment of prioritization and synthesis abilities
		Necessity of higher-order thinking beyond rote memorization
		Immersion in multimedia-assisted materials
	Perceived realism and validity of practice-based assessment	Clearer representation of clinical situations through multimedia materials
		Support for intuitive judgment and decision-making in realistic contexts
		Close connection between assessment and actual clinical practice
		Realistic reflection of clinical situations and practice-based experience
		Realistic assessment of clinical competence
		Practice-based evaluation reflecting actual job performance
	Linkage between simulation and clinical practice	Facilitation of problem solving through simulation experience
		Support for problem solving through clinical training experience
		Recognition of the importance of active engagement in clinical practice and simulation
Directions for enhancing the educational value of multimedia-assisted assessment	Educational value and learning effects of multimedia-assisted items	Ease of understanding and faster problem solving
		Intuitive comprehension of complex concepts through visualized materials
		Increased immersion and engagement during assessment
		Shift from text memorization to video- and image-based learning
	Establishing an educational foundation to expand the use of MAIs	Expansion of educational use of MAIs
		Need for standardized multimedia educational materials
		Improvement of accessibility and usability of multimedia materials
	Need for appropriate and complementary use of MAIs	Need to avoid excessive use of MAIs
		Necessity of complementary use of text-based items and MAIs

MAI, multimedia-assisted item.

## Footnotes

**Data availability**
Data files are available from https://doi.org/10.7910/DVN/IEIDXS Dataset 1. Binary item response data from the mock examination.

Dataset 2. Participant characteristics and post-test survey data.

## Supplementary materials

Supplement 1. Survey questionnaire.

Supplement 2. Focus group interview guide.

Supplement 3. Correlations of item parameters between paired multimedia-assisted and text-based items.

Supplement 4. Comparison of total scores and ability parameters.

Supplement 5. Comparison of item parameter estimates between the MT and TM groups.

Supplement 6. R script for item analysis using the irtQ package and data analysis outputs.

Supplement 7. Audio recording of the abstract.
