# Supplementary material for: Validity evaluation of a mock examination including multimedia-assisted items for the Korean Nursing Licensing Examination: a mixed-methods study with a counterbalanced design
Source: J Educ Eval Health Prof. 2026 Jul 21;23:21. doi: 10.3352/jeehp.2026.23.21 (PMC13575734; doi:10.3352/jeehp.2026.23.21)
Supplement: Supplementary file 1 [file jeehp-23-21-suppl1.docx]

**Supplement 1.** Survey questionnaire

**A. Pre-examination survey questionnaire**

1. Are you a 4th-year nursing student?

a. Yes (the survey will begin)

b. No (the survey will not begin and will be terminated)

2. Gender

a. Male

b. Female

3. Age

4. Region of your university

a. Seoul

b. Gyeonggi/Incheon

c. Gangwon

d. Gwangju/Jeolla

e. Busan/Ulsan/Daegu/Gyeongsang

f. Daejeon/Chungcheong/Sejong

g. Jeju

5. Prior to this exam, have you ever taken an exam that included multimedia-based items?

a. Yes → Go to Question 6

b. No → Go to Question 7

6. What type(s) of multimedia were used in the exam(s) you took prior to this exam? (Multiple responses allowed)

a. Image

b. Video

c. Audio

d. Other (	)

7. Please provide your mobile phone number. A token of appreciation will be sent to you at the provided number upon completion of your research participation. Additionally, your ID on the mock exam site will be set to your mobile phone number, and your initial password will be the last 4 digits of that number. (Example: If your phone number is 010-1234-5678, your ID will be 01012345678 and your password will be 5678)

**B. Post-examination survey questionnaire**

| Item | Rating scale | | | | |
| --- | --- | --- | --- | --- | --- |
|  | Strongly disagree | Disagree | Neutral | Agree | Strongly agree |
| 1. Multimedia-assisted items reflected clinical practice realistically. | ① | ② | ③ | ④ | ⑤ |
| 2. The multimedia materials provided in the items were helpful for problem solving. | ① | ② | ③ | ④ | ⑤ |
| 3. Multimedia-assisted items assess examinees’ abilities more accurately than text-based items. | ① | ② | ③ | ④ | ⑤ |
| 4. I did not experience major difficulty concentrating while solving multimedia-assisted items. | ① | ② | ③ | ④ | ⑤ |
| 5. The time provided to interpret and respond to the multimedia-assisted items was sufficient. | ① | ② | ③ | ④ | ⑤ |
| 6. I did not experience discomfort when using the screen/interface during the test. | ① | ② | ③ | ④ | ⑤ |

7. Did any of the exam items contain content that you had not studied?

a. Yes

b. No

8. To gain a more detailed understanding of examinees’ experiences with the mock exam, we plan to conduct online focus group interviews with 15 participants selected from among the examinees. Would you be willing to participate in a focus group interview at a later date? If you respond “Yes,” 15 participants will be randomly selected, and you will be contacted individually to schedule the focus group interview.

a. Yes

b. No
