# Supplementary material for: Validity evaluation of a mock examination including multimedia-assisted items for the Korean Nursing Licensing Examination: a mixed-methods study with a counterbalanced design
Source: J Educ Eval Health Prof. 2026 Jul 21;23:21. doi: 10.3352/jeehp.2026.23.21 (PMC13575734; doi:10.3352/jeehp.2026.23.21)
Supplement: Supplementary file 2 [file jeehp-23-21-suppl2.docx]

**Supplement 2.** Focus group interview guide

| No. | Interview question |
| --- | --- |
| 1 | What was most memorable while taking the test that included MAIs? |
| 2 | Compared with text-based items, what differences did you notice in the process of solving MAIs? |
| 3 | Please describe your process of solving the MAIs. |
| 3-1 | How did you understand the situation presented in the item? |
| 3-2 | How did you interpret the information given in the item? |
| 3-3 | Did the item feel similar to an actual clinical situation? |
| 4 | Which type of item do you think is more appropriate for assessing the job competency of newly graduated nurses: MAIs or text-based items? |
| 5 | Did what you learned in theory courses or clinical practice help you solve the MAIs? |
| 6 | If you were to take an examination using MAIs in the future, what kind of preparation or learning content do you think would be helpful? |
| 7 | Please share any other opinions you have about MAIs. |

MAIs, multimedia-assisted items.
