# Supplementary material for: Validity evaluation of a mock examination including multimedia-assisted items for the Korean Nursing Licensing Examination: a mixed-methods study with a counterbalanced design
Source: J Educ Eval Health Prof. 2026 Jul 21;23:21. doi: 10.3352/jeehp.2026.23.21 (PMC13575734; doi:10.3352/jeehp.2026.23.21)
Supplement: Supplementary file 3 [file jeehp-23-21-suppl3.docx]

**Supplement 3.** Correlations of item parameters between paired multimedia-assisted and text-based items

| Item parameter | r | P-value |
| --- | --- | --- |
| CTT difficulty | 0.94 | <0.001 |
| CTT discrimination (upper–lower discrimination index) | 0.92 | <0.001 |
| Corrected item–total correlation | 0.83 | <0.001 |
| 2PL difficulty (b parameter) | 0.84 | <0.001 |
| 2PL discrimination (a parameter) | 0.90 | <0.001 |

CTT, classical test theory; 2PL, 2-parameter logistic item response theory.
