# Supplementary material for: Validity evaluation of a mock examination including multimedia-assisted items for the Korean Nursing Licensing Examination: a mixed-methods study with a counterbalanced design
Source: J Educ Eval Health Prof. 2026 Jul 21;23:21. doi: 10.3352/jeehp.2026.23.21 (PMC13575734; doi:10.3352/jeehp.2026.23.21)
Supplement: Supplementary file 4 [file jeehp-23-21-suppl4.docx]

**Supplement 4.** Comparison of total scores and ability parameter

| Variable | MT group (n=256) | TM group (n=259) | Mean difference | t-value | P-value |
| --- | --- | --- | --- | --- | --- |
| Total score | 36.76±6.87 | 37.49±7.10 | –0.73 | –1.19 | 0.235 |
| Ability parameter | –0.07±0.88 | 0.07±0.92 | –0.15 | –1.83 | 0.068 |

Values are presented as mean±standard deviation unless otherwise stated. MT group: MAIs first, then text-based items; TM group: text-based items first, then MAIs.

MAIs, multimedia-assisted items.
