# Supplementary material for: Validity evaluation of a mock examination including multimedia-assisted items for the Korean Nursing Licensing Examination: a mixed-methods study with a counterbalanced design
Source: J Educ Eval Health Prof. 2026 Jul 21;23:21. doi: 10.3352/jeehp.2026.23.21 (PMC13575734; doi:10.3352/jeehp.2026.23.21)
Supplement: Supplementary file 5 [file jeehp-23-21-suppl5.docx]

**Supplement 5.** Comparison of item parameter estimates between the MT and TM groups (N=58 items)

| Parameter | MT group | TM group | Mean difference | t-value | P-value | r | P-value |
| --- | --- | --- | --- | --- | --- | --- | --- |
| CTT difficulty | 0.63±0.22 | 0.65±0.21 | –0.01 | –1.01 | 0.316 | 0.90 | <0.001 |
| CTT discrimination | 0.29±0.16 | 0.30±0.15 | –0.01 | –0.74 | 0.463 | 0.71 | <0.001 |
| Corrected item-total correlation | 0.21±0.09 | 0.23±0.09 | –0.02 | –1.35 | 0.181 | 0.51 | <0.001 |
| IRT difficulty (b) | 7.60±69.00 | 39.53±269.59 | –31.93 | –0.87 | 0.387 | –0.01 | 0.927 |
| IRT discrimination (a) | 0.65±0.34 | 0.86±0.74 | –0.21 | –2.39 | 0.020 | 0.45 | <0.001 |

Values are presented as mean±standard deviation unless otherwise stated. MT group: MAIs first, then text-based items; TM group: text-based items first, then MAIs.

CTT, classical test theory; IRT, item response theory; MAIs, multimedia-assisted items.
